# Intracellular calcium dynamics in cortical microglia responding to focal laser injury in the PC::G5-tdT reporter mouse

**DOI:** 10.3389/fnmol.2015.00012

**Published:** 2015-05-08

**Authors:** Amir Pozner, Ben Xu, Sierra Palumbos, J. Michael Gee, Petr Tvrdik, Mario R. Capecchi

**Affiliations:** ^1^Department of Human Genetics, University of UtahSalt Lake City, UT, USA; ^2^Department of Chemistry, University of UtahSalt Lake City, UT, USA; ^3^Howard Hughes Medical InstituteChevy Chase, MD, USA; ^4^Department of Bioengineering, University of UtahSalt Lake City, UT, USA

**Keywords:** calcium imaging, GECI, GCaMP5G, PC::G5-tdT, microglia, purinergic receptors

## Abstract

Microglia, the resident immune cells of the brain parenchyma, are highly responsive to tissue injury. Following cell damage, microglial processes redirect their motility from randomly scouting the extracellular space to specifically reaching toward the compromised tissue. While the cell morphology aspects of this defense mechanism have been characterized, the intracellular events underlying these responses remain largely unknown. Specifically, the role of intracellular Ca^2+^ dynamics has not been systematically investigated in acutely activated microglia due to technical difficulty. Here we used live two-photon imaging of the mouse cortex ubiquitously expressing the genetically encoded Ca^2+^ indicator GCaMP5G and fluorescent marker tdTomato in central nervous system microglia. We found that spontaneous Ca^2+^ transients in microglial somas and processes were generally low (only 4% of all microglia showing transients within 20 min), but baseline activity increased about 8-fold when the animals were treated with LPS 12 h before imaging. When challenged with focal laser injury, an additional surge in Ca^2+^ activity was observed in the somas and protruding processes. Notably, coherent and simultaneous Ca^2+^ rises in multiple microglial cells were occasionally detected in LPS-treated animals. We show that Ca^2+^ transients were pre-dominantly mediated via purinergic receptors. This work demonstrates the usefulness of genetically encoded Ca^2+^ indicators for investigation of microglial physiology.

## Introduction

Microglia are the principal immune cells in the brain. In their ramified, non-activated state, microglia exhibit small somas and elaborate, highly motile processes (Nimmerjahn et al., 2005). This high degree of motility facilitates the interaction of microglial bulbous endings with numerous physiological processes in the central nervous system, ranging from response to cellular damage (Davalos et al., 2005; Wake et al., 2009) to a role in synaptic pruning and response to neuronal activity (Paolicelli et al., 2011; Schafer et al., 2012; Dissing-Olesen et al., 2014; Eyo et al., 2014). The normal ramified morphology of microglia is dependent on the immune-privileged environment of the brain parenchyma and is difficult to reproduce *in vitro*, as serum and other factors present in the culture media cause immunological activation and subsequent changes in cell morphology and activity. Therefore, intravital imaging approaches that would limit tissue damage such as live two-photon microscopy are necessary for accurate investigations of cellular and physiological responses of microglia in the brain. Although synthetic dyes have been used widely for some applications, genetically encoded fluorescence markers afford significant advantages for intravital imaging experiments (Jung et al., 2000). To enable more detailed studies, we have recently developed a mouse reporter of cellular activity (PC::G5-tdT), which combines the use of a constitutively fluorescent protein (tdTomato) with the GCaMP5G intracellular Ca^2+^ indicator and displays high activity in microglia (Gee et al., 2014).

Ca^2+^ transients reveal underlying intracellular responses to extracellular signals in virtually all cell types of the nervous system (Berridge et al., 2003; Nedergaard et al., 2010; Grienberger and Konnerth, 2012). The information derived from Ca^2+^ signaling *in vivo* is particularly valuable in the studies of microglia, which are not electrically excitable and exceptionally difficult to load with synthetic dyes (Eichhoff et al., 2011; Garaschuk, 2013). As a consequence of limited technology, very little is known about the intracellular activity in microglia responding to physiological and pathophysiological brain processes. One striking example is the well-established paradigm of microglial processes responding to focal laser injury (Davalos et al., 2005; Haynes et al., 2006), which is well-characterized regarding the cell morphology of protruding processes, but poorly understood in terms of intracellular Ca^2+^ dynamics. To overcome this deficiency, we have used a newly generated *Aif1(Iba1)*-IRES-Cre mouse driver to express the PC::G5-tdT reporter in microglia and study the laser ablation paradigm with two-photon microscopy in anesthetized mice. Our results reveal new insights about the relationship between process motility, Ca^2+^ signaling and involvement of purinergic receptors.

## Materials and methods

### Animals

Generation of the PC::G5-tdT mouse line was described previously (Gee et al., 2014). The *Aif1(Iba1)*-IRES-Cre strain was generated by inserting the IRES-Cre-FRT-neo-FRT cassette into the 3′ untranslated region of the *Aif1* gene and will be described elsewhere. Additional details are available upon request. The *neo* selection marker was removed by breeding to FLP deleter and bred to homozygosity. All experimental animals were obtained by crossing homozygous PC::G5-tdT reporters with homozygous *Aif1*-IRES-Cre mice. All experiments were reviewed and approved by the University of Utah IACUC committee.

### Immunohistochemistry and confocal imaging

Adult mice used for immunohistochemistry were deeply anesthetized with Avertin (250 mg/kg body weight) then transcardially perfused with 4% paraformaldehyde (PFA) (EMS 15713) in 1 × PBS (pH 7.6). Following transfusion, brains were dissected, post-fixed in 4% PFA at room temperature, then processed through 10% sucrose in 1 × PBS at 4°C overnight followed by 30% sucrose in 1 × PBS until the brains sank. These brains were embedded in 2% gelatin (Sigma G2500) and 0.9% NaCl in a metal mold, flash frozen on a metal block cooled with liquid nitrogen. Brains were sectioned with a Leica CM1900 at 20 μm thickness, and mounted on SuperFrost Gold Plus microscopy slides (Fisher Scientific). The sections were incubated with primary antibodies, chicken anti-GFP (Aves GFP-1020) and rabbit anti-Iba1 (Wako 019-19741), diluted in Cyto-Q Immuno Diluent and Block (Innovex Biosciences). The sections were then incubated with secondary antibodies, goat anti-chicken AF488 (Life Technologies A11039) and goat anti-rabbit AF647 (Life Technologies A21244) diluted in Cyto-Q immune Diluent. The slides were washed with 1 × PBS and then mounted with Fluoromount-G (Southern Biotech). Slides were imaged with a Leica TCS SP5 confocal microscope. The images were analyzed and processed with Imaris 7.7.2 (Bitplane).

### Cranial window surgery and two-photon imaging

GCaMP5G-tdTomato labeled cells in cortical layers 1–3 were imaged by two-photon microscopy through a small craniotomy. Surgery was performed as previously described (Mostany and Portera-Cailliau, 2008). Briefly, 2–4 month old mice were anesthetized by inhalation of isoflurane (Vetone) (4% for induction, 1.5% for surgery and imaging). The anesthetized mice were placed on a 37°C heating pad (FHC, Bowdoin, ME) and the depth of anesthesia was evaluated by examination of pinch withdrawal, eyelid reflex, vibrissae movements, and respiration rate. After hair removal and disinfection with 10% povidone-iodine (Vetone), the scalp skin was removed and the skull membrane was scraped using a razor blade. The skull was thoroughly dried with a sterile cotton swab and a circle (~4 mm in diameter) over the visual or somatosensory cortex was thinned using a high-speed dental drill under a dissecting scope. To prevent damage of the underlying cortex by friction-induced heat, drilling was periodically interrupted to allow heat dissipation while saline was applied to the skull. A window in the skull was carefully opened with forceps keeping the dura intact, and a drop of sterile saline (Teknova) was applied to the exposed region. If needed, a GELFOAM (Pfizer) was applied to control bleeding. A thin 8 mm #0 coverslip was then glued to the skull with Cyanoacrylate (Superglue). One end of a flattened steel nail was cemented (A–M Systems) to the skull behind the window and the other end was inserted into a custom made holder to immobilize the head during imaging. Two-photon imaging was performed with a Prairie Technologies Ultima Multiphoton Microscopy System with a Chameleon Ti:Sapphire laser tuned to 920 nm and a 16× water-immersion objective (0.8 NA; Nikon) at a zoom of 4.3×. The maximal output of the laser power at the sample was kept low (<20 mW) to avoid unintended photo damage. Signal was acquired with GaAsP detectors, using a 490–560 nm bandpass filter for GCaMP5G (Ch3) and a 570–620 nm bandpass filter for tdTomato (Ch2). The maximum imaging depth was < 100 μm below the pial surface and sampling rate was 0.125 frames/s at 1024 × 1024 pixel resolution. Several recordings were acquired at 512 × 512 pixel resolution and 2 s per frame (0.5 Hz) rate (see Supplementary Figures 3E,F). PrairieView 5.2 software was used for image acquisition.

### Two-photon laser ablation

Focal laser injury was inflicted by focusing the two-photon laser beam onto a small area in the superficial cortex. The wavelength was set to 920 nm and laser power increased by 400–500%. A small area of 8 × 8 pixels was scanned at a frequency of 1 Hz for 90 s. The site of injury was visible as a bright auto-fluorescent sphere with a diameter of 10–15 μm.

### Drug administration

Lipopolysaccharide (LPS), from *Escherichia coli* strain 0111:B4 (Sigma, L4391) was dissolved in sterile saline and injected subcutaneously (50 μl, 1 mg/kg body weight) close to the midline of the lower lip. At 12 h, 24 h or 1 month following a single LPS injection, a craniotomy was performed in preparation for *in vivo* imaging. Pharmacological compounds were applied directly to the cortex on the intact dura prior to mounting the cranial window. Imaging was initiated 30 min after application and drugs were maintained in the preparation during the entire session. BAPTA-AM, PPADS (pyridoxal-5-phosphate-6-azophenyl-2′4-disulfonic acid) and bicuculline were purchased from Tocris. Stock solutions were diluted with sterile saline to a final concentration of 5 μg/ml, 5 mM, and 250 μM, respectively.

### Image processing and analysis

Image processing was performed with NIH ImageJ and Imaris (Bitplane) software. Focal drift was corrected using Imaris 7.7.2 prior to measurements of process motility or Ca^2+^ amplitudes. Process movement was analyzed with the filament-tracking algorithm included in the Imaris package. The distal end of a process was tracked as long as tdTomato was detectable through the 20 min imaging session. Care was taken to include only process ends that displayed directionality toward the injury site. Parameter settings were determined empirically and kept constant for all analyses of process motility, including: Filament Quality > 40, Max Distance = 3, Max Gap Size = 5, Track Duration > 5 min and Track Displacement Length > 5 min. Process motility was quantified for each cell by averaging the velocity of each process belonging to that cell. Image animations were generated with iMovie. For calcium transient detection in laser injury experiments, the data was first carefully examined in Imaris in slow motion. The spatial location of each potential transient was manually delineated as a region of interest (ROI) including all pixels perceived to be associated with the event. The mean fluorescence intensity F_j_ of all pixels belonging to an ROI was computed for each frame j. The resulting J-frame time series *F*_1_…*F*_J_ was then plotted and analyzed in Microsoft Excel and Igor Pro (WaveMetrics), using the MultiPeak Fitting 2 Package and Wave Stats. The lowest mean intensity value in each time series was set to 0, baseline was fitted with a cubic polynomial function and potential peaks were detected with the multi-peak fitting algorithm. The baseline was then subtracted and only the transients with amplitudes greater than 2 standard deviations above the mean baseline fluorescence were used in analysis. The temporal boundaries of each transient were determined as the frames containing the first (F_j_^*^) and the last (F_j_^*^ + *K* − 1) values above the local baseline *F*_0_ (defined for each transient as F_j_^*^ − 1). The highest *F*_k_ was designated as the maximum value for that transient (*F*_max_). The amplitude of a transient (Δ*F*) was measured in the original trace as *F*_max_–*F*_0_. The instance of a transient was defined as the elapsed time between the initiation of imaging and the frame containing F_j_^*^. Please refer to Supplementary Figure 3 for additional information.

### Statistical analysis

Unless otherwise stated, all results are reported as mean ± SEM and statistical tests were considered significant when *p* < 0.05. Statistical calculations (Student's *t*-test, ANOVA) were performed with Microsoft Excel.

## Results

### Genetic labeling of cortical microglia with GCaMP5G-IRES-tdTomato

**Figure 1:**
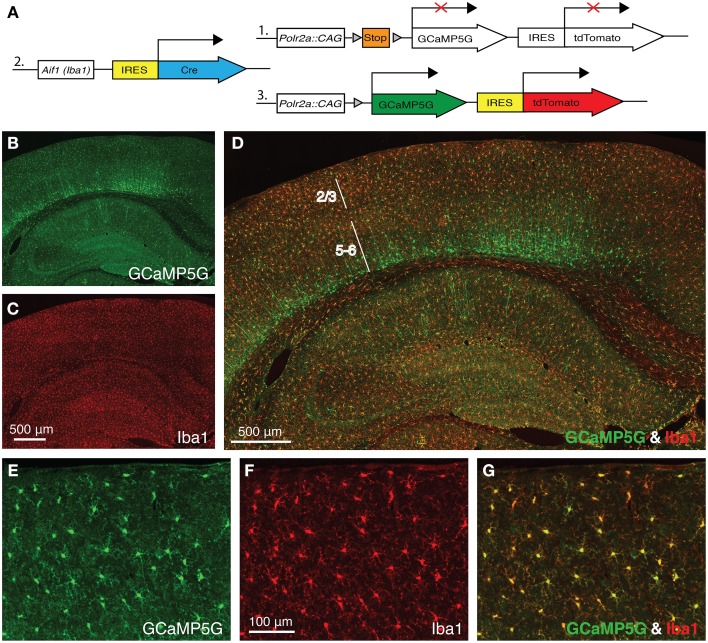
***Aif1*-IRES-Cre driver labels Iba1-positive microglia. (A)** A schematic diagram of the PC::G5-tdT allele (1) crossed with *Aif1*-IRES-Cre (2). Expression of IRES-Cre at the *Aif1* locus results in the expression of GCaMP5G and tdTomato following Cre-mediated excision of the STOP cassette (3). **(B–D)** A coronal section of *Aif1*-IRES-Cre; PC::G5-tdT brain stained with anti-GFP (GCaMP5G) and anti-Iba1 antibodies. **(B)** The section was imaged specifically in the green channel (GCaMP5G expression), **(C)** red channel (Iba1 expression) and **(D)** overlay of green and red channels. The PC::G5-tdT allele is expressed in all *Aif1* lineage cells, including layer 5/6 cortical neurons. Layers 2/3 and 5–6 are indicated. **(E–G)** Coronal sections of somatosensory cortex layer 2/3 region of *Aif1*-IRES-Cre; PC::G5-tdT brains stained with anti-GFP and anti-Iba1 antibodies. **(E)** GCaMP5G expression, **(F)** endogenous Iba1 staining and **(G)** Overlay. PC::G5-tdT was co-expressed in all Iba1-positive cells studied in these experiments. Adult animals (6–8 weeks) were used for immunohistochemistry in **(B-G)**.

Allograft inflammatory factor 1 (*Aif1*), also known as ionized Ca^2+^-binding adapter molecule 1 (*Iba1*), is specifically expressed in microglia in the adult brain. In order to express the GCaMP5G Ca^2+^ indicator (Akerboom et al., 2012) and the tdTomato fluorescence marker in cortical microglia, we crossed a newly generated *Aif1*-IRES-Cre driver to the PC::G5-tdT reporter (Gee et al., 2014) (Figure 1A). When we stained the brains of the progeny with anti-GFP antibody (cross-reacting with GCaMP5G) and anti-Iba1 antibody, virtually all cortical microglia were co-labeled (Figures 1D,G). Reproducibly, the *Aif1*-IRES-Cre driver also labeled a number of cortical neurons in layer 6, few hippocampal pyramidal neurons in CA1 and very sparsely in other regions of the brain (Figures 1B–D) suggesting a brief transcriptional activation of the *Aif1* gene during early corticogenesis. Nonetheless, in the superficial cortical layers 1–3, which are relevant for *in vivo* imaging through cranial windows, the vast majority of labeled cells are microglia (Figures 1E–G). Out of 644 GCaMP5G-positive cells counted, 627 were also co-labeled with anti-Iba1 antibody and 17 were GCaMP5G positive only (2.6%).

### *In vivo* imaging and focal laser stimulation of genetically labeled microglia

Previously, Ca^2+^ dynamics in adult cortical microglia were studied using single cell electroporation of synthetic Ca^2+^ indicators, such as OGB-1 (Eichhoff et al., 2011). The electroporation procedure was not considered to affect microglia physiology, since OGB-1-labeled cells maintained their ramified morphology for at least 1 h and retained their ATP chemotactic response. Using this method, it was demonstrated that 22% of resting microglia exhibited spontaneous Ca^2+^ transients in the course of a 15 min imaging period.

**Figure 2:**
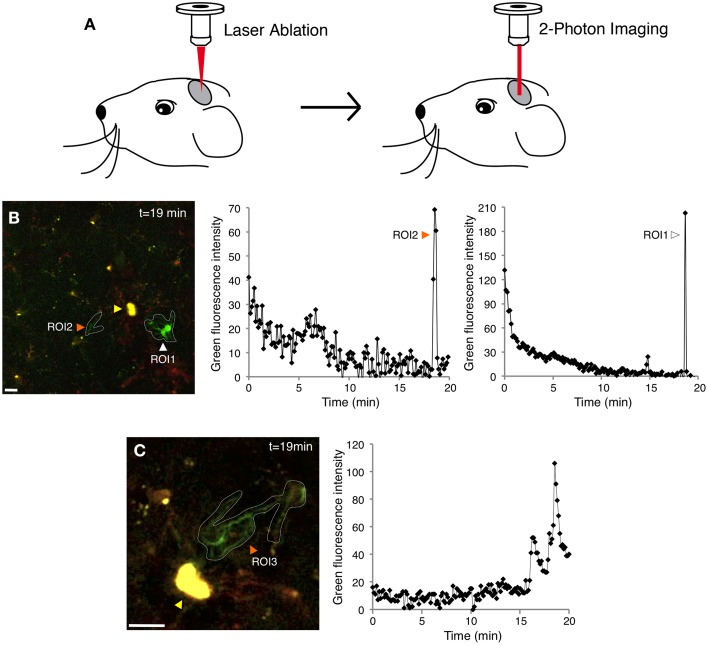
**Simultaneous two-photon imaging of tdTomato and GCaMP5G signals in cortical microglia. (A)** Cartoon demonstrating the two-photon microscopy approach used to image microglial Ca^2+^ responses after laser inflicted focal injury in layer 2/3 of the somatosensory or visual cortex. **(B)** GCaMP5G fluorescence remains stable, allowing long imaging sessions (Supplementary Movie 2). A recording of green fluorescence intensity in ROI1 (white arrowhead) shows a large Ca^2+^ transient in the soma and processes 19 min after the laser injury. Another recording in ROI2 (orange arrowhead) shows a Ca2+ transient localized mainly to the microglial processes. The corresponding tdTomato fluorescence time series for this experiment is shown in Supplementary Figure 2B and Supplementary Movie 1. **(C)** A different example of a process-restricted Ca^2+^ transient. Variability in branch spiking was often observed in the same cell (Supplementary Movie 3). ROI; Region of Interest. Yellow arrowheads indicate the laser injury sites. Scale bars: 10 μm.

**Figure 3:**
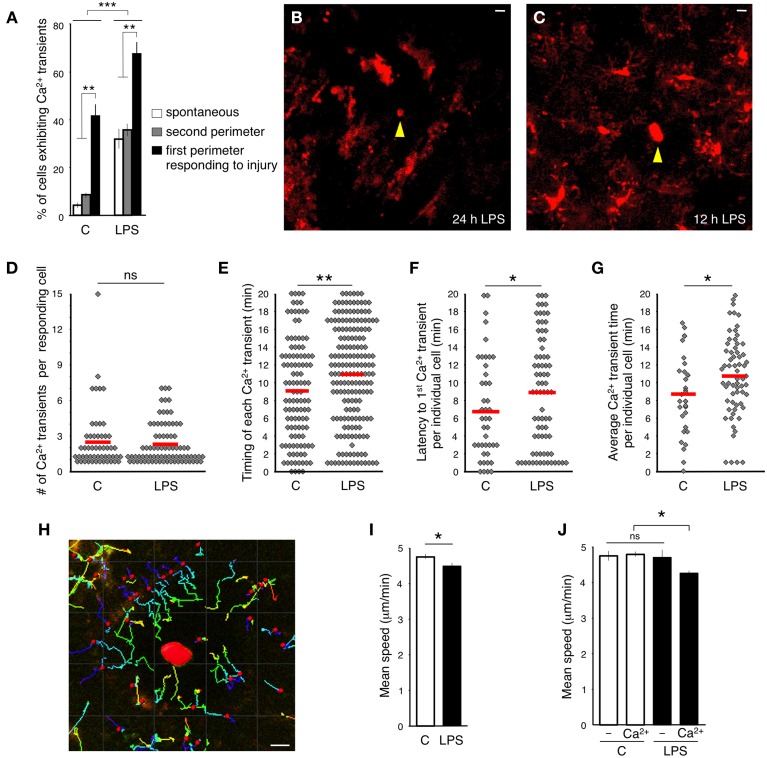
**Effect of systemic peripheral inflammation on intracellular calcium dynamics in microglia. (A)** Percentage of microglia exhibiting Ca^2+^ transients in response to focal laser injury and LPS induced peripheral inflammation. White bars show the percentage of cells exhibiting spontaneous Ca^2+^ transients. Gray bars specify the percentage of 2nd perimeter microglia positive for Ca^2+^ transients. Black bars illustrate the percentage of Ca^2+^ positive 1st perimeter microglia. We define 1st perimeter microglia as the circle of microglial cells that are most proximal to the laser ablation site. Their processes always spread toward the injury. 2nd perimeter microglia have their cell bodies located farther away, behind the 1st perimeter microglia. 2nd perimeter microglial processes do not spread toward the injury in our experiments. **(B)** A representative image of inflammation-activated microglia with amoeboid-like appearance. Focal laser injury (yellow arrowhead) was induced 24 h after subcutaneous LPS administration and imaged immediately after the ablation. **(C)** Twelve hour after the injection, LPS-primed microglia maintained normal ramified morphology. Focal laser injury (yellow arrowhead) was induced 12 h after LPS administration. **(D)** A graph illustrating the distribution of the number of Ca^2+^ transients in responding cells during the entire imaging session. No significant (ns) differences were detected between control and LPS-primed cells. The red lines indicate the means. **(E)** Time between injury and each individual Ca^2+^ transient emerging in responding cells. **(F)** Latency between the time of injury and the first Ca^2+^ transient observed in a specific responding cell. **(G)** Latency from the time of injury to the average emergence time of all Ca^2+^ transients detected per one responding cell. **(H)** An example of tracking the microglial process movement with Imaris software. The injury site is marked in red. **(I)** Average speed of process protrusion was significantly reduced in LPS-primed microglia. **(J)** No process speed difference was found between LPS-primed microglia not exhibiting Ca^2+^ transients and control cells. In contrast, the mean process speed was significantly reduced in LPS-primed Ca^2+^-positive microglia. Bar graphs are mean ± SEM from *n* = 3 mice (>10 cells per mouse), (>18 cells per mouse in **I–J**). ^*^p < 0.05, ^**^p < 0.005 by Student's *t*-test, ^***^p < 0.02 by a 2 × 3 factor ANOVA (treatments × groups). Scale bars: 10 μm.

Here we adapted two-photon laser scanning microscopy through a cranial window (Figure 2A and Materials and Methods) to record tdTomato and GCaMP5G signals in Iba1-positive cells in the mouse cortex (Supplementary Figures 1–3 and Figure 2). In this model, only 4% of resting microglia exhibited at least one spontaneous Ca^2+^ transient during a 20 min recording session (Supplementary Figure 1 and Figure 3A) (5/116, *n* = 3 mice), suggesting that OGB-1 electroporation does affect physiological properties of resting microglia within the 1 h experimental time frame. The use of genetically encoded Ca^2+^ indicators suggests that resting microglia exhibit even fewer spontaneous Ca^2+^ transients than formerly thought.

Next, we tested the Ca^2+^ response to focal injury by inflicting a small laser ablation. In accordance with previously published data (Davalos et al., 2005), microglial processes close to the site of injury thickened their bulbous endings and spread toward the injured site (Supplementary Movie 1). The cortical damage evoked Ca^2+^ transients in 42% of the responding GCaMP5G-expressing microglia (in the first perimeter around the injury, 51/123, *n* = 3 mice) during 20-min long imaging session (Figure 3A). Again, in a comparable experiment probing synthetic dye-loaded microglia with micropipette-provoked neuronal damage, 100% of the cells displayed Ca^2+^ transients within 15 min after stimulus (Eichhoff et al., 2011), which is an apparent exaggeration compared to the genetically encoded reporter.

In our experiments, the cells located farther away from the ablation (2nd perimeter) did not demonstrate a directional response toward the injury and did not exhibit significantly increased incidence of Ca^2+^ transients (Figure 3A and Supplementary Movies 2, 3). The majority of injury-induced Ca^2+^ transients (>80%) arose in the microglia processes only, while the remainder occurred both in the processes and cell bodies (Figures 2B,C and Supplementary Movies 2–3).

### Systemic peripheral inflammation increases the frequency of calcium transients in brain microglia

Peripheral administration of gram-negative bacterial cell-wall component LPS initiates peripheral inflammation effects. The interaction of LPS with macrophage Toll-like receptors provokes production of proinflammatory cytokines including TNFα, IL-1β, and IL-6 (Jaffer et al., 2010). Subsequently, the cytokines cross the blood brain barrier via an endocrine-like mechanism and activate microglia (Maier et al., 1998; Lee et al., 2010; Ousman and Kubes, 2012). We sought to evaluate the microglial Ca^2+^ response in the brains of mice that were subjected to peripheral inflammation. In agreement with previous observations (Gyoneva et al., 2014), imaging 24 h after subcutaneous LPS administration to the lower lip revealed that all microglia altered their morphology and phenotype, adopting an activated state. Cells transformed into an amoeboid morphology by retracting their processes, which become fewer and much thicker, and by increasing the size of their soma (Figure 3B). Accordingly, microglia displayed an amoeboid-like movement in the vicinity of the site of insult (Supplementary Movie 4). Moreover, activated microglia did not show Ca^2+^ transients during the 20 min long imaging sessions.

Interestingly, when we performed imaging experiments 1 month after a single LPS injection to the lower lip, microglia returned to their resting ramified structure and resumed a typical response to focal brain injury spreading their processes toward the site of insult. In contrast, these formerly activated cells did not resume normal frequency of Ca^2+^ transients in the first perimeter of the injury (4.9%, 4/82, *n* = 2 mice) (Supplementary Movie 5). Hence, the long-term inhibitory effect of a single peripheral immune challenge on microglial Ca^2+^ signaling is in line with the previously shown long-lasting microglial “memory” marked by persistent increase of Iba1 immunoreactivity (Kondo et al., 2011).

When we imaged microglia 12 h after subcutaneous LPS administration, we found that the cells had not yet acquired an amoeboid-like activated appearance (Figure 3C). Moreover, these cells displayed an unusually high frequency of spontaneous Ca^2+^ transients (34%, 45/131, *n* = 3 mice) (Figure 3A). We termed these cells LPS-primed microglia. Significantly more primed microglia responding to focal laser injury displayed Ca^2+^ transients (67%, 77/115, *n* = 3 mice) (Figure 3A). The number of Ca^2+^ transients in more distal primed microglia was similar to resting primed cells (37%, 68/182, *n* = 3 mice) (Figure 3A). Most non-primed microglia exhibited only one Ca^2+^ transient in the processes in close apposition to the injury site (110 of 182; 60.4%), suggesting that in naïve microglia, Ca^2+^ activity is triggered in the vicinity of the lesion, either due to the high concentration of a diffusible factor or by the direct physical contact with the lesion. However, the distribution of Ca^2+^ transients in primed microglia was different and displayed an approximate 1:1 ratio (85, or 47.5%, showed transients before the contact and 94, or 52.5%, after the contact), suggesting that primed-microglia in the state of systemic peripheral inflammation are either more sensitized, or subjected to increased concentrations of diffusible factors, or both. There were no significant differences in number of Ca^2+^ transients per injury-evoked cell between LPS-primed and non-primed microglia, within 20 min after injury (Figure 3D). However, the latency between injury induction and the emergence of the Ca^2^ transients was significantly delayed in LPS-primed injury-evoked microglia (Figures 3E–G).

The amplitude of Ca^2+^ transients (Δ*F*) in LPS-primed microglia was not significantly different from control (LPS: 153.3 ± 19.2 vs. control: 128.9 ± 18.4; *p* = 0.193, Student's *t*-test; *n* = 22 transients in each group) nor was the difference in transient duration (LPS; 26.9 ± 1.9 s, vs. control: 25.0 ± 1.7 s; *p* = 0.237, Student's *t*-test; *n* = 22 transients per group).

Next, we measured the velocity of the processes protruding toward the focal injury. The tdTomato signal allowed for continuous monitoring of the bulbous endings, despite gradual photo bleaching (Supplementary Figures 1C,D), using the Imaris tracking algorithm (see the Materials and Methods, Figure 3H). In line with previous findings (Gyoneva et al., 2014), we determined that the average peak velocity of process extension was decreased in the LPS-primed microglia compared to controls (LPS; 4.53 ± 0.12 μm/min, vs. control; 4.80 ± 0.08 μm/min; *p* = 0.038, Student's *t*-test; *n* > 58 cells) (Figure 3I). Of note, this decrease was primarily accounted for by the LPS-primed microglia that exhibited Ca^2+^ transients (Figure 3J). In subsequent studies, we used LPS-primed microglia as an experimental baseline for additional pharmacological analyses.

### PPADS and BAPTA-AM have differential effects on microglial calcium transients and process motility

**Figure 4:**
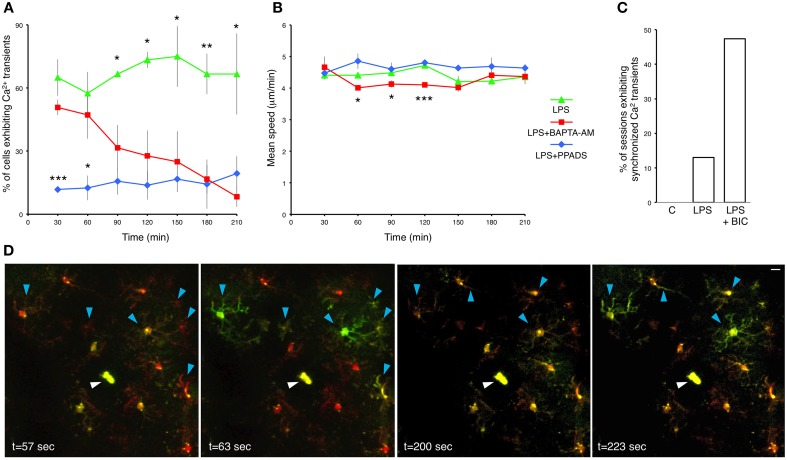
**Pharmacological interrogation of calcium responses in LPS-primed microglia. (A)** Percentage of LPS-primed microglia exhibiting Ca^2+^ transients in response to focal laser injury as a function of time. BAPTA-AM (5 μg/ml) and PPADS (5 mM) were topically applied through the craniotomy 12 h after subcutaneous LPS administration. Two focal laser injuries were induced half an hour later, and were immediately followed by a 20 min long imaging session. Successive injury/imaging sessions were taken in 30 min intervals. Upper asterisks refer to statistical significance between LPS and LPS+BAPTA-AM treatments. Lower asterisks refer to statistical significance between LPS+BAPTA-AM and LPS+PPADS treatments. **(B)** Average speed of microglial processes as a function of time after BAPTA-AM and PPADS application. **(C)** A bar graph illustrating the fraction of imaging sessions which include synchronized microglial Ca^2+^ transients during 20 min recordings after focal laser injury. C-control: untreated mice. LPS: primed mice 12 h post-subcutaneous LPS administration. LPS+BIC: 12 h LPS-primed mice treated with topical application of bicuculline (250 μM). **(D)** Time lapse images from a representative 20 min recording session showing synchronized microglial Ca^2+^ transients traversing across the entire imaging field. This particular example shows two distinct waves at *t* = 63 s and *t* = 223 s. Blue arrowheads indicate cell bodies and processes of the microglia demonstrating synchronized Ca^2+^ waves. White arrowheads indicate the laser injury sites. Data are mean ± SEM (*n* = 3 mice, 4–10 cells for each time point per mouse). ^*^p < 0.05, ^**^p < 0.01, ^***^p < 0.005 by Student's *t*-test. Scale bar: 10 μm.

The relationship between microglial Ca^2+^ transients and process motility in not well-understood. Therefore, we examined the dependence of process extension velocity on intracellular Ca^2+^ by clamping intracellular Ca^2+^ with cell-permeable BAPTA-AM. BAPTA-AM is converted in the cytoplasm to BAPTA, an intracellular Ca^2+^ chelator (Negulescu et al., 1989). As expected, topical application of BAPTA-AM through the craniotomy in LPS-primed mice gradually reduced the frequency of microglial Ca^2+^ transients (Figure 4A). Hence, Consistent with previous observations (Eichhoff et al., 2011), microglial Ca^2+^ transients are dependent on the availability of intracellular Ca^2+^. Moreover, BAPTA-AM application significantly slowed down the process velocity at multiple time points post-administration (e.g., at 60 min, LPS; 4.41 ± 0.09 μm/min, vs. BAPTA-AM; 4.01 ± 0.07 μm/min; *p* = 0.0214, Student's *t*-test; *n* = 3 mice) (Figure 4B). However, after 2.5 h the differences were no longer significant. Our data imply that the velocity of microglial process protrusion toward the site of insult is dependent on permissive basal Ca^2+^ levels.

We cannot exclude the possibility that our experiments encompass various interactions between microglia and astrocytes. It has been shown that BAPTA-AM impedes microglia reaction to injury by blocking the astrocyte polarization response (Kim and Dustin, 2006). Injury induced Ca^2+^ increase in astrocytes has been shown to induce ATP secretion, which, in turn, activates purinergic receptors in microglia (Verderio and Matteoli, 2001). When we applied the P2X and P2Y antagonist PPADS, it reduced the frequency of Ca^2+^ transients in LPS-primed microglia with high efficiency (Figure 4A). In fact, PPADS down-regulated Ca^2+^ transients more rapidly than BAPTA-AM. Conversely, PPADS did not significantly affect the microglial process movement (Figure 4B). Hence, our results do not strongly support the role of purinergic receptors in process motility, but identify them as major mediators of intracellular Ca^2+^ transients.

### Synchronous wave-like calcium activity in LPS-primed microglia

Coordinated Ca^2+^ activity in cortical astrocytic networks has been documented (Hirase et al., 2004b), but as yet there has been no evidence of synchronized Ca^2+^ activity in microglia *in vivo*. In our spontaneous, LPS-untreated preparations, synchronized Ca^2+^ activity was never detected (Figure 4C). However, we occasionally recorded synchronized Ca^2+^ transients in LPS-primed injury-responding microglia (Figures 4C,D and Supplementary Movie 6). About 13% (3 of 23) of 20 min recording sessions post-focal laser injury conducted on LPS-primed mice exhibited synchronized Ca^2+^ transients (Figure 4C). Bicuculline, a GABA_A_ antagonist, attenuates inhibitory synaptic input onto neurons, tipping the excitation/inhibition balance in favor of excitation. As a result, bicuculline has been used to study neuronal hyperactivity in models of epilepsy and Alzheimer's disease (Schwartz and Bonhoeffer, 2001; Busche et al., 2008). In previous studies, microglial Ca^2+^ waves have not been detected *in vivo* after bicuculline administration (Eichhoff et al., 2011; Brawek and Garaschuk, 2013). In contrast, we observed frequent synchronized Ca^2+^ transients in microglia following bicuculline application (47%, 9 of 19, *n* = 3 mice) (Figure 4C). Some of the waves spread across the entire imaging field (Figure 4D and Supplementary Movie 6), while others were more confined (Supplementary Movie 7). Interestingly, these microglial waves often occurred concomitantly with transient brain motion (Supplementary Movies 7–8). We also observed an increase in cerebral blood flow after bicuculline administration as previously described (Hirase et al., 2004a), which typically manifested as brief expansions of blood capillaries (Supplementary Movie 8). Synchronized microglial Ca^2+^ activity following bicuculline-administration likely reflects epileptiform activity in local neuronal networks. Neuronal release of diffusible factors, such as ATP, activate purinergic receptors expressed by microglia and result in increased Ca^2+^ wave frequency and process motility (Sieger et al., 2012).

## Discussion

Recording of intracellular signaling in microglia has been complicated by technical difficulties with dye loading and subsequent amplification of these distortions due to high immune reactivity of these cells. Although the use of cell culture systems and invasive cell labeling techniques provided important initial insights, these findings need to be validated under *in vivo* imaging conditions with endogenous indicators. Here, we targeted the endogenously encoded Ca^2+^ indicator GCaMP5G to all Iba1-positive microglia *in vivo* and used this novel mouse model to study intracellular Ca^2+^ signaling in both control and LPS-challenged microglia. We demonstrate that the PC::G5-tdT reporter mouse provides useful readout for both cell motility and intracellular Ca^2+^ activity in minimally invasive preparations.

In contrast with high motility of the processes, spontaneous intracellular Ca^2+^ transients in unchallenged, resting microglia are very infrequent in the anesthetized brain. In our model, only 4% of recorded microglia showed transients during the imaging period of 20 min, which is in conflict with similar experiments performed with electroporated dyes published by Eichhoff et al. In fact, the percentage of active microglia published by them (22%) is more akin to the situation we observe in LPS-primed microglia (34%), and implies that cell electroporation brings about microglial cell activation. Although our sampling frequency was limited to 0.125 Hz, it is unlikely that any transients went undetected since the average event duration was >20 s, and higher frame rate recordings did not reveal any short-duration events (Supplementary Figures 3E,F).

P2ry12 is the principal purinergic receptor expressed by microglia, along with P2ry13 (Hickman et al., 2013). Microglial response to focal injury in *P2ry12* mutants is attenuated (Haynes et al., 2006) and purinergic antagonists such as PPADS have been claimed to affect the motility of microglial processes responding to injury (Davalos et al., 2005). In our hands, PPADS application dramatically and rapidly decreased the percentage of cells displaying Ca^2+^ rises (Figure 4A), unlike the studies performed with synthetic dyes (Eichhoff et al., 2011). However, this PPADS-induced suppression of Ca^2+^ activity did not affect the velocity of bulbous end protrusion (Figure 4B). Thus, our data does not support the view that transient Ca^2+^ rises elicited via P2ry12 receptors contribute to process motility. When we applied BAPTA-AM, however, both the frequency of Ca^2+^ transients and velocity of protrusions were significantly decreased, implying that basal intracellular Ca^2+^ levels are still required for full motility in responding microglia.

The role for Ca^2+^ rises in microglia *in vivo* remains to be fully clarified. Studies performed on microglial cells *in vitro* strongly suggest that transient rises in Ca^2+^ concentration mediate the release of nitric oxide, certain cytokines, and chemokines, which are important for the recruitment and activation of various cell types (Hoffmann et al., 2003). It is of high importance to establish the neurological correlates of intracellular Ca^2+^ dynamics in these cells *in vivo*, with potential implications for conditions where microglia are thought to play a role, such as epilepsy (Devinsky et al., 2013), Alzheimer's disease (Aguzzi et al., 2013) or schizophrenia (Mizoguchi et al., 2014). Development of more powerful genetic tools is therefore highly desirable and will facilitate progress in this field.

## Author contributions

PT conceived the study, PT and AP designed the experiments, AP performed *in vivo* imaging and data analysis, BX generated the *Aif1*-IRES-Cre driver, JG created the PC::G5-tdT mouse line, SP performed immunohistochemistry and confocal imaging, PT and AP wrote the manuscript with editing by JG, MC provided resources and proofread the manuscript.

### Conflict of interest statement

The authors declare that the research was conducted in the absence of any commercial or financial relationships that could be construed as a potential conflict of interest.

## References

[B1] Aguzzi A. Barres B. A. Bennett M. L. (2013). Microglia: scapegoat, saboteur, or something else? Science 339, 156–161. 10.1126/science.1227901 23307732 PMC4431634

[B2] Akerboom J. Chen T. W. Wardill T. J. Tian L. Marvin J. S. Mutlu S.. (2012). Optimization of a GCaMP calcium indicator for neural activity imaging. J. Neurosci. 32, 13819–13840. 10.1523/JNEUROSCI.2601-12.2012 23035093 PMC3482105

[B3] Berridge M. J. Bootman M. D. Roderick H. L. (2003). Calcium signalling: dynamics, homeostasis and remodelling. Nat. Rev. Mol. Cell Biol. 4, 517–529. 10.1038/nrm1155 12838335

[B4] Brawek B. Garaschuk O. (2013). Microglial calcium signaling in the adult, aged and diseased brain. Cell Calcium 53, 159–169. 10.1016/j.ceca.2012.12.003 23395344

[B5] Busche M. A. Eichhoff G. Adelsberger H. Abramowski D. Wiederhold K. H. Haass C.. (2008). Clusters of hyperactive neurons near amyloid plaques in a mouse model of Alzheimer's disease. Science 321, 1686–1689. 10.1126/science.1162844 18802001

[B6] Davalos D. Grutzendler J. Yang G. Kim J. V. Zuo Y. Jung S.. (2005). ATP mediates rapid microglial response to local brain injury *in vivo*. Nat. Neurosci. 8, 752–758. 10.1038/nn1472 15895084

[B7] Devinsky O. Vezzani A. Najjar S. De Lanerolle N. C. Rogawski M. A. (2013). Glia and epilepsy: excitability and inflammation. Trends Neurosci. 36, 174–184. 10.1016/j.tins.2012.11.008 23298414

[B8] Dissing-Olesen L. Ledue J. M. Rungta R. L. Hefendehl J. K. Choi H. B. MacVicar B. A. (2014). Activation of neuronal NMDA receptors triggers transient ATP-mediated microglial process outgrowth. J. Neurosci. 34, 10511–10527. 10.1523/JNEUROSCI.0405-14.2014 25100586 PMC6802598

[B9] Eichhoff G. Brawek B. Garaschuk O. (2011). Microglial calcium signal acts as a rapid sensor of single neuron damage *in vivo*. Biochim. Biophys. Acta 1813, 1014–1024. 10.1016/j.bbamcr.2010.10.018 21056596

[B10] Eyo U. B. Peng J. Swiatkowski P. Mukherjee A. Bispo A. Wu L.-J. (2014). Neuronal hyperactivity recruits microglial processes via neuronal NMDA receptors and microglial P2Y12 receptors after status epilepticus. J. Neurosci. 34, 10528–10540. 10.1523/JNEUROSCI.0416-14.2014 25100587 PMC4200107

[B11] Garaschuk O. (2013). Imaging microcircuit function in healthy and diseased brain. Exp. Neurol. 242, 41–49. 10.1016/j.expneurol.2012.02.009 22370088

[B12] Gee J. M. Smith N. A. Fernandez F. R. Economo M. N. Brunert D. Rothermel M.. (2014). Imaging activity in neurons and glia with a Polr2a-based and cre-dependent GCaMP5G-IRES-tdTomato reporter mouse. Neuron 83, 1058–1072. 10.1016/j.neuron.2014.07.024 25155958 PMC4156920

[B13] Grienberger C. Konnerth A. (2012). Imaging calcium in neurons. Neuron 73, 862–885. 10.1016/j.neuron.2012.02.011 22405199

[B14] Gyoneva S. Davalos D. Biswas D. Swanger S. A. Garnier-Amblard E. Loth F.. (2014). Systemic inflammation regulates microglial responses to tissue damage *in vivo*. Glia 62, 1345–1360. 10.1002/glia.22686 24807189 PMC4408916

[B15] Haynes S. E. Hollopeter G. Yang G. Kurpius D. Dailey M. E. Gan W.-B.. (2006). The P2Y12 receptor regulates microglial activation by extracellular nucleotides. Nat. Neurosci. 9, 1512–1519. 10.1038/nn1805 17115040

[B16] Hickman S. E. Kingery N. D. Ohsumi T. K. Borowsky M. L. Wang L.-C. Means T. K.. (2013). The microglial sensome revealed by direct RNA sequencing. Nat. Neurosci. 16, 1896–1905. 10.1038/nn.3554 24162652 PMC3840123

[B17] Hirase H. Creso J. Buzsaki G. (2004a). Capillary level imaging of local cerebral blood flow in bicuculline-induced epileptic foci. Neuroscience 128, 209–216. 10.1016/j.neuroscience.2004.07.002 15450368

[B18] Hirase H. Qian L. Barthó P. Buzsáki G. (2004b). Calcium dynamics of cortical astrocytic networks *in vivo*. PLoS Biol. 2:E96. 10.1371/journal.pbio.0020096 15094801 PMC387267

[B19] Hoffmann A. Kann O. Ohlemeyer C. Hanisch U.-K. Kettenmann H. (2003). Elevation of basal intracellular calcium as a central element in the activation of brain macrophages (microglia): suppression of receptor-evoked calcium signaling and control of release function. J. Neurosci. 23, 4410–4419. 12805281 10.1523/JNEUROSCI.23-11-04410.2003 PMC6740788

[B20] Jaffer U. Wade R. G. Gourlay T. (2010). Cytokines in the systemic inflammatory response syndrome: a review. HSR Proc. Intensive Care Cardiovasc. Anesth. 2, 161–175. 23441054 PMC3484588

[B21] Jung S. Aliberti J. Graemmel P. Sunshine M. J. Kreutzberg G. W. Sher A.. (2000). Analysis of fractalkine receptor CX(3)CR1 function by targeted deletion and green fluorescent protein reporter gene insertion. Mol. Cell. Biol. 20, 4106–4114. 10.1128/MCB.20.11.4106-4114.2000 10805752 PMC85780

[B22] Kim J. V. Dustin M. L. (2006). Innate response to focal necrotic injury inside the blood-brain barrier. J. Immunol. 177, 5269–5277. 10.4049/jimmunol.177.8.5269 17015712

[B23] Kondo S. Kohsaka S. Okabe S. (2011). Long-term changes of spine dynamics and microglia after transient peripheral immune response triggered by LPS *in vivo*. Mol. Brain 4:27. 10.1186/1756-6606-4-27 21682853 PMC3138393

[B24] Lee S. Zhao Y. Q. Ribeiro-Da-Silva A. Zhang J. (2010). Distinctive response of CNS glial cells in oro-facial pain associated with injury, infection and inflammation. Mol. Pain 6:79. 10.1186/1744-8069-6-79 21067602 PMC2992508

[B25] Maier S. F. Goehler L. E. Fleshner M. Watkins L. R. (1998). The role of the vagus nerve in cytokine-to-brain communication. Ann. N. Y. Acad. Sci. 840, 289–300. 10.1111/j.1749-6632.1998.tb09569.x 9629257

[B26] Mizoguchi Y. Kato T. A. Horikawa H. Monji A. (2014). Microglial intracellular Ca(2+) signaling as a target of antipsychotic actions for the treatment of schizophrenia. Front. Cell. Neurosci. 8:370. 10.3389/fncel.2014.00370 25414641 PMC4220695

[B27] Mostany R. Portera-Cailliau C. (2008). A craniotomy surgery procedure for chronic brain imaging. J. Vis. Exp. 12:e680. 10.3791/680 19066562 PMC2582844

[B28] Nedergaard M. Rodríguez J. J. Verkhratsky A. (2010). Glial calcium and diseases of the nervous system. Cell Calcium 47, 140–149. 10.1016/j.ceca.2009.11.010 20045186

[B29] Negulescu P. A. Reenstra W. W. Machen T. E. (1989). Intracellular Ca requirements for stimulus-secretion coupling in parietal cell. Am. J. Physiol. 256, C241–C251. 2465690 10.1152/ajpcell.1989.256.2.C241

[B30] Nimmerjahn A. Kirchhoff F. Helmchen F. (2005). Resting microglial cells are highly dynamic surveillants of brain parenchyma *in vivo*. Science 308, 1314–1318. 10.1126/science.1110647 15831717

[B31] Ousman S. S. Kubes P. (2012). Immune surveillance in the central nervous system. Nat. Neurosci. 15, 1096–1101. 10.1038/nn.3161 22837040 PMC7097282

[B32] Paolicelli R. C. Bolasco G. Pagani F. Maggi L. Scianni M. Panzanelli P.. (2011). Synaptic pruning by microglia is necessary for normal brain development. Science 333, 1456–1458. 10.1126/science.1202529 21778362

[B33] Schafer D. P. Lehrman E. K. Kautzman A. G. Koyama R. Mardinly A. R. Yamasaki R.. (2012). Microglia sculpt postnatal neural circuits in an activity and complement-dependent manner. Neuron 74, 691–705. 10.1016/j.neuron.2012.03.026 22632727 PMC3528177

[B34] Schwartz T. H. Bonhoeffer T. (2001). *In vivo* optical mapping of epileptic foci and surround inhibition in ferret cerebral cortex. Nat. Med. 7, 1063–1067. 10.1038/nm0901-1063 11533712

[B35] Sieger D. Moritz C. Ziegenhals T. Prykhozhij S. Peri F. (2012). Long-range Ca2+ waves transmit brain-damage signals to microglia. Dev. Cell 22, 1138–1148. 10.1016/j.devcel.2012.04.012 22632801

[B36] Verderio C. Matteoli M. (2001). ATP mediates calcium signaling between astrocytes and microglial cells: modulation by IFN-gamma. J. Immunol. 166, 6383–6391. 10.4049/jimmunol.166.10.6383 11342663

[B37] Wake H. Moorhouse A. J. Jinno S. Kohsaka S. Nabekura J. (2009). Resting microglia directly monitor the functional state of synapses *in vivo* and determine the fate of ischemic terminals. J. Neurosci. 29, 3974–3980. 10.1523/JNEUROSCI.4363-08.2009 19339593 PMC6665392

