# Supplementary material for: Intracellular calcium dynamics in cortical microglia responding to focal laser injury in the PC::G5-tdT reporter mouse
Source: Front Mol Neurosci. 2015 May 8;8:12. doi: 10.3389/fnmol.2015.00012 (PMC4424843; doi:10.3389/fnmol.2015.00012)
Supplement: Supplementary file 9 [file DataSheet1.PDF]

Supplementary Material

Intracellular calcium dynamics in cortical microglia responding to focal laser injury in the PC::G5-tdT reporter mouse

Amir Pozner[1,2], Ben Xu[1,4], Sierra Palumbos[1], J. Michael Gee[3], Petr Tvrdik[1,]*, Mario R. Capecchi[1,3]

[1]University of Utah, Department of Human Genetics, Salt Lake City, Utah 84112, USA.

[2]University of Utah, Department of Chemistry, Salt Lake City, Utah 84112, USA.

[3]University of Utah, Department of Bioengineering, Salt Lake City, Utah 84112, USA.

[4]Howard Hughes Medical Institute, 4000 Jones Bridge Road, Chevy Chase, MD 20815-6789, USA.

* **Correspondence:** Petr Tvrdik, University of Utah, Department of Human Genetics, Salt Lake City, Utah, USA. petr.tvrdik@genetics.utah.edu

1. Supplementary Figures

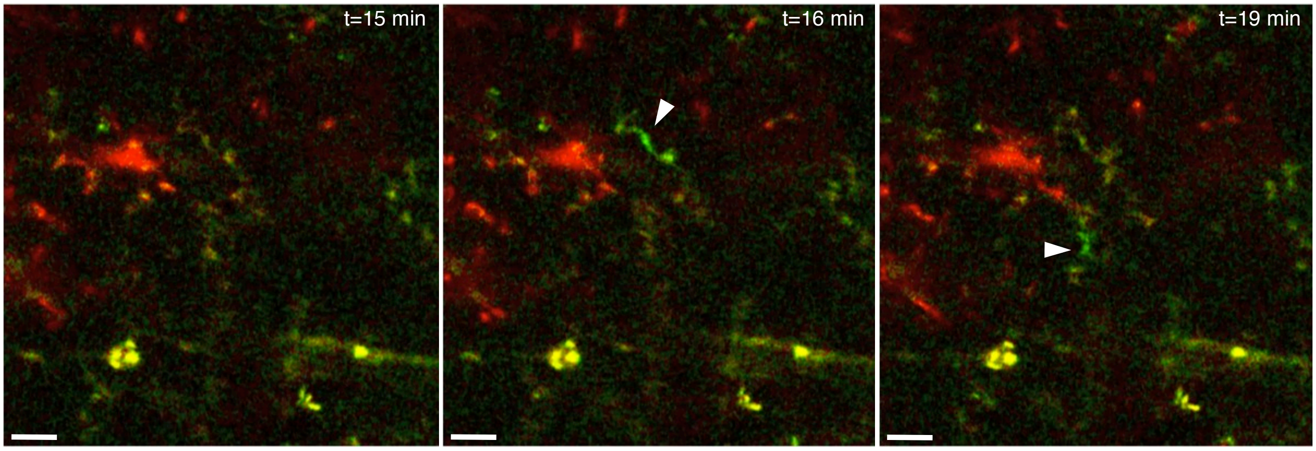

SUPPLEMENTARY FIGURE 1. Representative images of tdTomato and GCaMP5G fluorescence in Iba1-PC::G5-tdT microglia. Arrowheads indicate two rare spontaneous Ca^{2+} transients in naïve surveillant microglia. Scale bars: 10 µm.

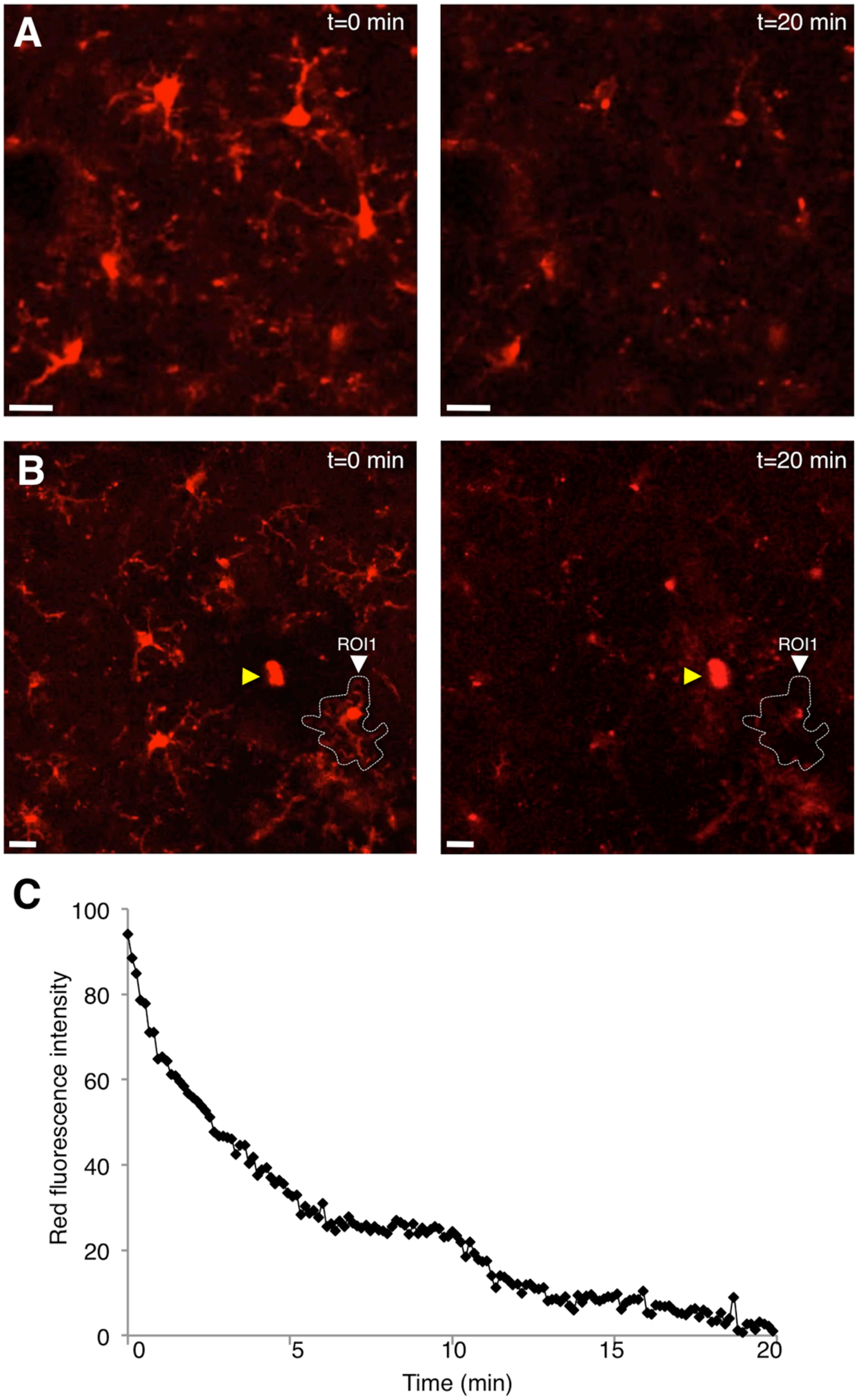

SUPPLEMENTARY FIGURE 2. Optical signal attenuation of tdTomato. (A and B) Representative images of tdTomato fluorescence in Iba1-PC::G5-tdT microglia showing photobleaching typically observed with this fluorescence marker during long imaging sessions. **(A)** Imaging of control surveillant microglia. **(B)** Imaging after focal laser ablation. Tissue damage (yellow arrowheads) is visible as a bright autofluorescent sphere. Left panels; initial frames captured at the beginning of image acquisition. Right panels; image frames captured after 20 min. This experiment is also shown in **Figure 2B** and **Supplementary Movies 1-2**. **(C)** Recording of tdTomato fluorescence in the soma and processes of a single cell shown in panel B (ROI1, white arrowheads) over the time course of 20 min. Scale bars: 10 µm.

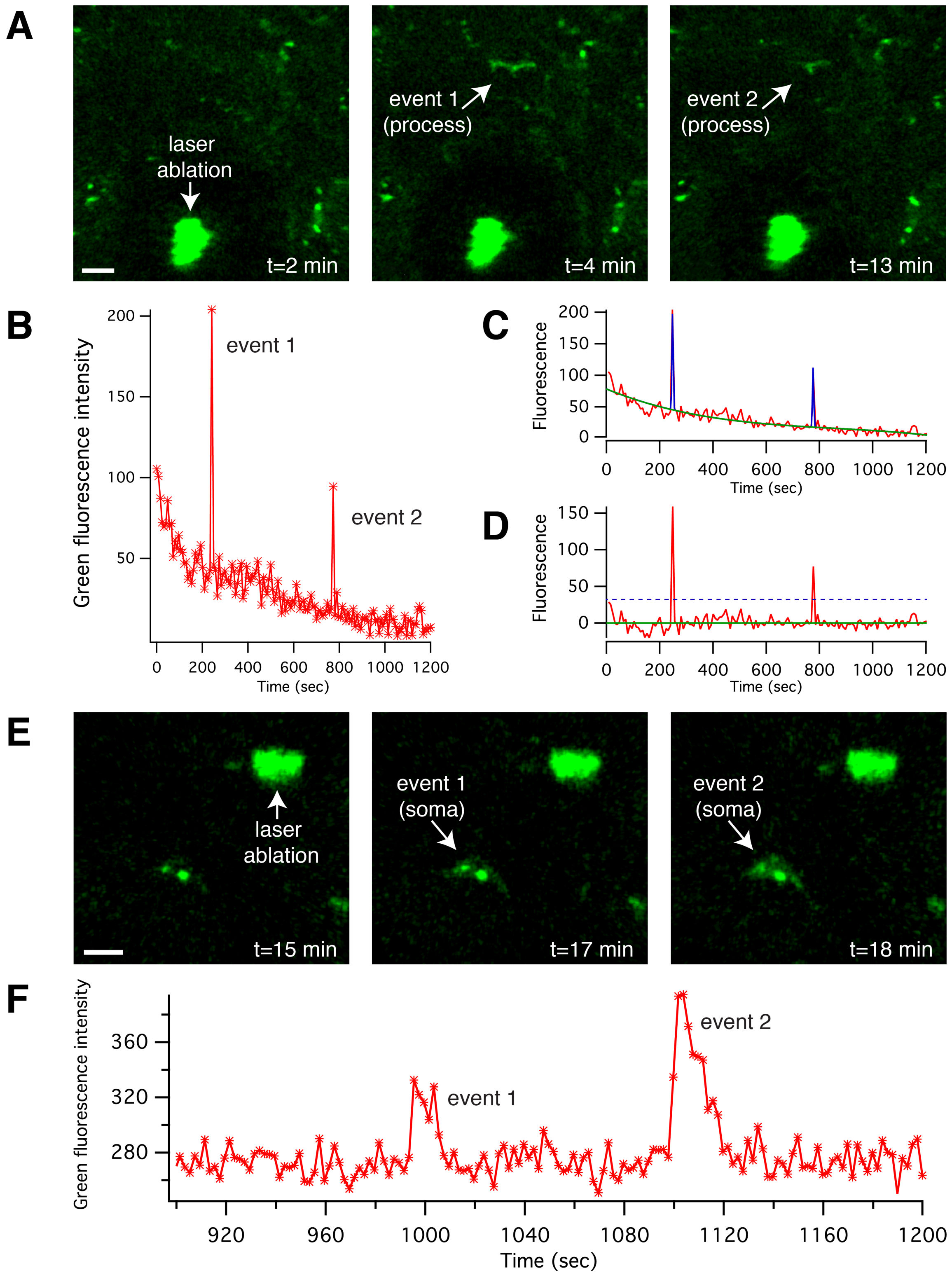

SUPPLEMENTARY FIGURE 3. GCaMP5G signal analysis. (A) Image acquisition conditions used in this study were 1024x1024 pixel resolution at 8 s per frame (0.125 Hz). The laser ablation scar and Ca^{2+} calcium transients in a microglial process are indicated in a dataset obtained with this resolution. **(B)** An unprocessed fluorescence intensity trace of the events identified in **(A)**. **(C)** Raw fluorescence signal fitted with cubic polynomial baseline (green line) and a multi-peak detection algorithm (blue line). **(D)** The baseline-subtracted signal superimposed with the detection threshold used in

this study (blue dotted line; two standard deviations above the baseline). **(E)** Recording obtained with a faster scanning speed (512x512 pixels, 2 s per frame [0.5 Hz]). **(F)** The last 5 minutes of unprocessed fluorescence signal recorded from a microglial soma in this higher-speed laser ablation experiment. The duration of Ca^{2+} transients in this recording was found to be 12 and 20 s, respectively (6 and 10 frames), consistent with the rest of the study. Scale bars: 10 µm.

2. Legends for Supplementary Movies

Supplementary Movie 1. Two-photon imaging of tdTomato signal in microglia. A time series corresponding to **Figure 2B**, showing a cell response to localized laser injury. The tissue damage is evident as a bright auto-fluorescent sphere. Surrounding microglia immediately extend their processes and invade the damaged area. Only the red fluorescence channel is displayed, demonstrating photobleaching of the tdTomato signal during the 20-min imaging session.

Supplementary Movie 2. Simultaneous two-photon imaging of GCaMP5G and tdTomato in microglia. A time series corresponding to **Figure 2B**, displaying an overlay of the green and red channels from the experiment shown in **Supplementary Movie 1**. The tissue damage is evident as a bright yellow auto-fluorescent sphere. Note a Ca^{2+} transient at t = 19 min, localized to the process of the cell in left bottom corner. At t = 20 min, the right bottom cell exhibits a strong Ca^{2+} transient in both cell soma and processes.

Supplementary Movie 3. Two-photon recording of an extensive calcium transient in responding microglia. A time series corresponding to **Figure 2C**, demonstrating a sizeable Ca^{2+} transient in injury-responding microglia. Note the intense green fluorescence in the upper right cell, beginning at t = 17 min.

Supplementary Movie 4. Morphology and intracellular dynamics of activated microglia. Fully activated microglia 24 h after subcutaneous LPS administration show an aberrant response to laser injury. Cells have enlarged somas and retracted processes, display amoeboid-like, non-directional movements and do not generate Ca^{2+} transients.

Supplementary Movie 5. Microglial dynamics one month after LPS injection. One month after subcutaneous LPS administration, microglia display normal ramified morphology and typical process protrusion toward the site of insult. However, the cells fail to generate Ca^{2+} transients.

Supplementary Movie 6. Synchronized calcium activity in LPS-primed microglia. This time series corresponds to **Figure 4D**. 12 h LPS-primed microglia execute typical process extension toward the sites of insult. Note two successive waves of synchronized Ca^{2+} transients at t = 1 min, and t = 4 min.

Supplementary Movie 7. Calcium dynamics of LPS-primed, bicuculline-treated microglia. This time series shows 12 h LPS-primed microglia after topical application of bicuculline. Note robust, coordinated Ca^{2+} transients in adjacent microglia as their processes form a spherical containment around the site of insult, beginning at t = 12 min. The wave of synchronized microglial Ca^{2+} activity occurs concomitant with an abrupt tremor of the mouse brain.

Supplementary Movie 8. A blood flow surge in LPS-primed, bicuculline treated brain. A time series demonstrating 12 h LPS-primed microglia after topical application of bicuculline. Brain tremor is a well-documented consequence of topical bicuculline administration due to an increase in cerebral blood flow. Note a wave of synchronized microglial Ca^{2+} transients at t = 10 min, followed by a brief expansion of a nearby blood capillary (bottom right) at t = 11 min.
